# Hataedock Treatment Has Preventive Therapeutic Effects in Atopic Dermatitis-Induced NC/Nga Mice under High-Fat Diet Conditions

**DOI:** 10.1155/2016/1739760

**Published:** 2016-05-24

**Authors:** Ho-Yeol Cha, Sang-hyun Ahn, Jin-Hong Cheon, In-Sik Park, Jin-Tack Kim, Kibong Kim

**Affiliations:** ^1^Department of Korean Pediatrics, Hospital of Korean Medicine, Pusan National University, 20 Geumo-ro, Mulgeum-eup, Yangsan-si, Gyeongsangnam-do 50612, Republic of Korea; ^2^Research Institute for Korean Medicine, Pusan National University, 49 Pusandaehak-ro, Mulgeum-eup, Yangsan-si, Gyeongsangnam-do 50612, Republic of Korea; ^3^Department of Anatomy, College of Korean Medicine, Dongguk University, Seokjang-dong, Gyeongju-si, Gyeongsangbuk-do 38066, Republic of Korea; ^4^Department of Korean Pediatrics, School of Korean Medicine, Pusan National University, 49 Pusandaehak-ro, Mulgeum-eup, Yangsan-si, Gyeongsangnam-do 50612, Republic of Korea

## Abstract

This study investigated the preventive therapeutic effects of Hataedock (HTD) treatment on inflammatory regulation and skin protection in AD-induced NC/Nga mice under high-fat diet conditions. Before inducing AD, the extract of* Coptidis Rhizoma *and* Glycyrrhiza uralensis *was administered orally to the 3-week-old mice. After that, AD-like skin lesions were induced by applying DNFB. All groups except the control group were fed a high-fat diet freely. We identified the effects of HTD on morphological changes, cytokine release and the induction of apoptosis through histochemistry, immunohistochemistry, and TUNEL assay. HTD downregulated the levels of IL-4 and PKC but increased the levels of LXR. HTD also suppressed the mast cell degranulation and release of MMP-9, Substance P. The levels of TNF-*α*, p-I*κ*B, iNOS, and COX-2 were also decreased. The upregulation of inflammatory cell's apoptosis is confirmed by our results as increase of apoptotic body and cleaved caspase-3 and decrease of Bcl-2. HTD also reduced edema, angiogenesis, and skin lesion inflammation. Our results indicate HTD suppresses various inflammatory response on AD-induced mice with obesity through the regulation of Th2 differentiation and the protection of lipid barrier. Therefore, HTD could be used as an alternative and preventive therapeutic approach in the management of AD.

## 1. Introduction

Atopic dermatitis (AD) is a chronic and relapsing inflammatory skin disease with a typical distribution of pruritic skin lesions. As with any other inflammatory skin disease, it greatly affects the quality of life of patients [1, 2]. Many studies have supported the definition of AD as a complex trait, in that interactions between genes and environmental factors and the interplay between multiple genes contribute to disease manifestation [3].

Traditionally, it was thought that the primary pathogenic mechanism of AD was initiated largely due to immune dysfunction, with the key roles played by Th1/Th2 cell dysregulation, IgE production, dendritic cell signaling, and mast cell hyperactivity, leading to pruritic, inflammatory dermatosis, and the secondary disruption of the epidermal barrier [4–6].

Recently, the importance of environmental factors during infancy and early childhood in the expression of AD has become well-recognized. It was suggested that the immune and respiratory systems are relatively immature during infancy and that they continue to develop during early childhood [7]. In addition, it was suggested that allergic diseases have their origins in early life and that the priming of the immune system starts in utero [8]. Previous studies have found that obesity is associated with defective skin barrier function [9, 10]. In addition, obesity results in a chronic low-grade inflammatory condition that may directly contribute to inflammatory pathways in AD [11]. Although the precise link between obesity and AD has not been well established, adiposity is believed to induce systemic inflammation, which may negatively influence the immature immune system and atopic disorders [12]. Based on these studies, we can conclude that early life is a crucial period of development for the immune system and that excess fat may influence the pathogenesis or expression of allergic diseases among susceptible infants and children.

Within traditional Korean Medicine, AD is thought to be caused by children being influenced by “heat” during fetal development, which is referred to as “fetal heat” [13]. This “fetal heat” is influenced by maternal diet or mentality during pregnancy. Therefore, traditional Korean Medicine treats AD with “heat-clearing” medicines to reduce accumulated heat in the skin [14]. Hataedock (HTD) treatment is a “heat-clearing” treatment that dispels “fetal heat” and the meconium collected by the fetus via orally administering herbal extracts to a newborn baby. In our previous study, HTD treatment alleviated inflammatory skin damages in NC/Nga mice through regulating of inflammation and downregulation of protein kinase C (PKC) and Th2 cytokines, which are involved in the initial steps of AD development [15, 16].

An extract of* Coptidis Rhizoma* and* Glycyrrhiza uralensis* is traditionally used in HTD treatment. Recent studies have indicated that* Coptidis Rhizoma*, a kind of classical heat-clearing and detoxifying herb according to traditional Korean pharmacology, has antihyperglycemia, antihyperlipidemia, antihypertension, anti-inflammatory, and antioxidant effects [17]. It is also known that* Coptidis Rhizoma* and its main active compound, berberine, improve glucose and lipid metabolism disorders and have antiobesity activities [18, 19].* Glycyrrhiza uralensis* is used to treat several inflammatory disorders, enhance the activity of other ingredients, reduce toxicity, and improve flavor [20]. The antioxidant and anti-inflammatory activities of flavonoids separated from* Glycyrrhiza uralensis* have been reported in recent years [21, 22]. The combination of these effects may lead to enhanced lipid metabolism and reduced skin inflammation in AD.

Based on this background, we evaluated the preventive therapeutic effects of HTD treatment on inflammatory regulation and skin protection in AD-induced NC/Nga mice fed a high-fat diet.

## 2. Materials and Methods

### 2.1. Preparation of HTD Herb Extract

The procedure used to manufacture the herb extract for HTD treatment was as follows: 100 g of* Coptidis Rhizoma* and 100 g of* Glycyrrhiza uralensis* were decocted in 1,000 mL of distilled water for 3 hours and then filtered; after concentrating this mixture to 50 mL under reduced pressure using a rotary evaporator, the filtrate was freeze-dried. We obtained 31 g of the extract (yield: 15.5%) for use.

### 2.2. Animal and AD Induction

Male 3-week-old NC/Nga mice (13–15 g each) were obtained from Central Lab Animal Inc. (Seoul, Korea). The mice were divided into four groups (*n* = 10 per group) as follows: the normal feeding group (Ctrl group), high-fat diet group (HF group), high-fat diet and AD-induced with no treatment group (HDE group), and high-fat diet and AD-induced with HTD treatment group (HTT group). In the HTT group, 3-week-old mice were given HTD treatment; they were given the extract of* Coptidis Rhizoma* and* Glycyrrhiza uralensis* orally (20 mg/kg) on days 1, 2, and 3. To induce AD-like skin lesions, the back regions of the mice were stripped, and 1 mL of 5% sodium dodecyl sulfate (SDS) (Sigma-Aldrich, St. Louis, MO, USA) was rubbed on the back of each mouse 20 times using a cotton swab to remove the lipid lamella of the stratum corneum. On day 28, the mice were sensitized via exposure to 100 *μ*L of 1% dinitrofluorobenzene (DNFB) in acetone/olive oil (4 : 1). On days 35 and 42, the mice were challenged with 2% DNFB 100 *μ*L. On day 45, the mice were deeply anesthetized with sodium pentobarbital and killed. Mice in the HF, HDE, and HTT groups except the control group were fed a high-fat diet (fat, 60%; carbohydrate, 20%; protein, 20%; DIO DIET, USA) freely for the experimental period.

**Figure 1:**
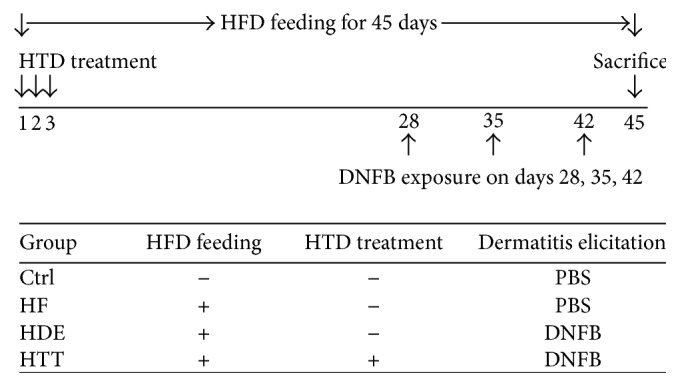
Experimental design. Before inducing AD, the extract of* Coptidis Rhizoma* and* Glycyrrhiza uralensis*, which is traditionally used in HTD treatment, was administered orally to the HTT group on days 1, 2, and 3. Mice were challenged by DNFB on days 28, 35, and 42. During the experimental period, all groups except the Ctrl group were fed a high-fat diet freely. Ctrl: normal feeding, HF: high-fat diet, HDE: high-fat diet and untreated AD-induced, HTT: high fat diet and Hataedock treated AD-induced, HFD: high-fat diet, HTD: Hataedock, PBS: phosphate-buffered saline, and DNFB: dinitrofluorobenzene.

All animal experiments were approved by the Institutional Animal Care and Use Committee of Pusan National University (IACUC number: PNU-2014-0732). We followed the NIH Guide for the Care and Use of Laboratory Animals throughout this study. The experimental design is summarized in Figure 1.

### 2.3. Fingerprinting Analysis

High-performance liquid chromatography- (HPLC-) based fingerprinting was performed with an Agilent 1200 Series HPLC System (Agilent Technologies, Santa Clara, CA, USA), binary solvent delivery pump (G1312A), autosampler (G1329A), column oven (G1316A), diode array detector (DAD; G1315D), vacuum degasser (G1322A), and Capcell PAKMGII C18 column (3.0 × 150 mm, 3.0 *μ*m; Shiseido, Tokyo, Japan). The flow rate of the column was set at 0.6 mL/min, the temperature was maintained at 35°C, and the injection volume was set at 15 *μ*L. The mobile phase consisted of 0.5% formic acid in water (v/v; A) and acetonitrile (B) with the following linear gradient profile: initiation 5 min, 2% B, 12 min, 10% B, 20 min, 25% B, 27 min, 25% B, 25 min, 80% B, 37 min, 80% B, 40 min, 30% B, and 45 min, 2% B. A standard solution containing Palmatine, Berberine (ChemFaces, Wuhan, China), Liquritin, and Liquiritigenin (Sigma-Aldrich, USA) was prepared by dissolving these compounds in distilled water (10 mg/100 mL). The solution was filtered through a 0.45 *μ*m syringe filter, after which HPLC was performed.

**Figure 2:**
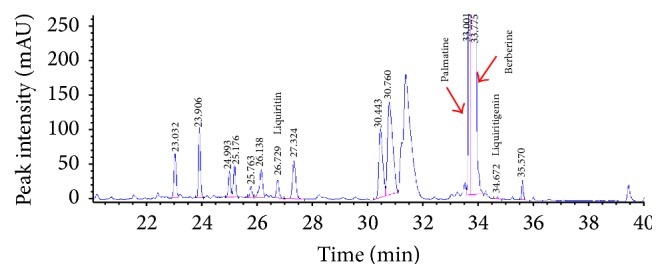
The HPLC analysis of the extract of* Coptidis Rhizoma* and* Glycyrrhiza uralensis*. Palmatine was detected at approximately 33.001 minutes, Berberine was detected at approximately 33.775 minutes, Liquritin was detected at approximately 26.729 minutes, and Liquiritigenin was detected at approximately 34.672 minutes. HPLC: High-performance liquid chromatography.

To identify the constituents of the herb extract used for HTD treatment (the extract of* Coptidis Rhizoma* and* Glycyrrhiza uralensis*) in the study, we conducted HPLC fingerprinting. The standard constituents of our component analysis of the extract were Palmatine, Berberine, Liquritin, and Liquritigenin. The HPLC analysis is shown in Figure 2.

### 2.4. Tissue Process and Histochemistry

After the mice were sacrificed, dorsal skins were obtained and fixed in 10% NBF at room temperature for 24 h and embedded in paraffin for serial sectioning (5 *μ*m).

To investigate histological changes such as epithelial hyperplasia, capillary distribution, and collagen fiber distribution, we performed Masson's trichrome staining, which is used to detect collagen fibers and collagen deposition. The samples were fixed using Bouin's fluid (50–60°C) for 1 hr. The picric acid was then removed with 70% ethanol. The samples were incubated in Weigert's iron hematoxylin working solution for 10 min to stain the nuclei, and then, the collagen fibers were stained blue with Biebrich scarlet-acid fuchsin solution and phosphomolybdic-phosphotungstic acid for 15 min each and aniline blue solution for 5 min.

To investigate the distribution and morphological changes of the mast cells that were activated by neuropeptide, we performed histochemical staining with Luna's stain. We stained the mast cell granules using an aldehyde fuchsin solution for 30 minutes and Weigert's iron hematoxylin working solution for 10 minutes and then counterstained in methyl orange solution for 5 minutes.

To investigate changes in the lipid lamella in the stratum corneum, we used the oil red O staining method. To create frozen sections for lipid staining, the samples were fixed with 10% NBF and 10% formol-calcium. We immersed the sample in a cryoprotection solution of 30% sucrose and made the frozen sections 10 mm in width by freezing microtome (Microm, Germany). After that, we placed the slices in oil red O for 10 minutes to drain off the water, counterstained with Harris hematoxylin for 2 minutes, rinsed in distilled water, then mounted the sample with glycerin jelly, and observed the slices.

### 2.5. Immunohistochemistry

The skin slices were steeped in proteinase K solution (20 *μ*g/mL) to undergo proteolysis for 5 minutes. The proteolysed slices were incubated in blocking serum (10% normal goat serum) for 4 hours. Then, the slices were incubated with goat anti-LXR (1 : 200, Santa Cruz Biotec, USA), goat anti-PKC (1 : 100, Santa Cruz Biotec, USA), goat anti-IL-4 (1 : 100, Santa Cruz Biotec, USA), goat anti-Substance P (1 : 100, Santa Cruz Biotec, USA), goat anti-MMP-9 (1 : 200, Santa Cruz Biotec, USA), goat anti-TNF-*α* (1 : 100, Santa Cruz Biotec, USA), goat anti-p-I*κ*B (1 : 500, Santa Cruz Biotec, USA), goat anti-iNOS (1 : 200, Santa Cruz Biotec, USA), goat anti-COX-2 (1 : 200, Santa Cruz Biotec, USA), goat anti-Bcl-2 (1 : 100, Santa Cruz Biotec, USA), and goat anti-cleaved caspase-3 (1 : 100, Santa Cruz Biotec, USA), all of which are primary antibodies, for 72 hours in a 4°C humidified chamber. Next, the slices were linked with biotinylated rabbit anti-goat IgG (1 : 100, Santa Cruz Biotec), which is a secondary antibody, for 24 hours at room temperature. After the slices were exposed to the secondary antibody, an avidin biotin complex kit (Vector Lab, USA) was applied for 1 hour at room temperature. Finally, the slices were developed with 0.05 M tris-HCl buffer solution (pH 7.4), which consisted of 0.05% 3, 3′-diaminobenzidine and 0.01% HCl, and then counterstained with hematoxylin.

### 2.6. TUNEL Assay

To investigate apoptosis, a TUNEL assay was performed using an* in situ* apoptosis detection kit (Apoptag, Intergen, USA). We carried out proteolysis using proteinase K for 5 minutes and then applied equilibration buffer for 5 seconds. The proteolysed slices were added strength TdT enzyme (36 *μ*L TdT enzyme : 72 *μ*L reaction buffer). Then, the slices were incubated in a humidified chamber at 37°C for 1 hour and then agitated for 10 minutes in strength stop/wash buffer. Next, the slices were treated with anti-digoxigenin peroxidase and DAB for 1 hour. Finally, we observed the sections counterstained with eosin using an optical microscope.

### 2.7. Image Analysis and Statistical Analysis

To produce numerical data from our immunohistochemistry, an image analysis was performed using image Pro Plus (Media cybernetics, USA). In image analysis of our 400x magnification exposure photography, the positive reacted particle as pixel cells (80–100 intensity range) was counted in 10 randomly selected fields of each group (total pixel cells 100,000,000 or 1,000,000 by various results of immunohistochemistry condition such as nonspecific structure and artificiality). The data were presented as the means ± standard error. The statistical significances of the differences were analyzed with SPSS software (SPSS 23, SPSS Inc., USA), using a one-way ANOVA and Levene's (LSD) test with a significance level of *p* < 0.01.

## 3. Results

### 3.1. The Regulation of Th2 Differentiation

**Figure 3:**
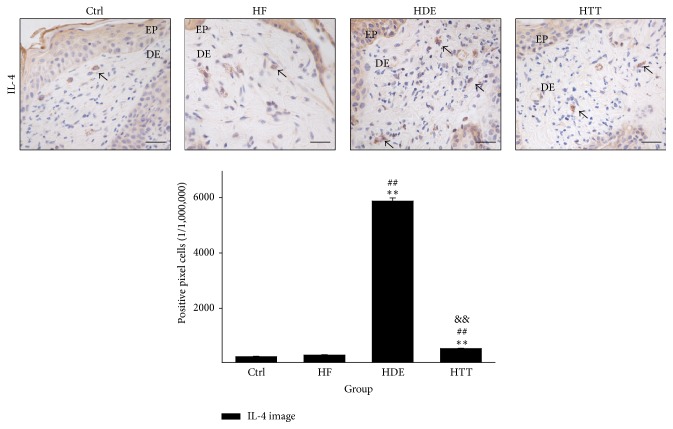
The regulation of Th2 differentiation. IL-4-positive reaction (arrow indicates dark brown) decreased in the HTT group compared with the HDE group (IL-4 immunohistochemistry; bar size, 50 *μ*m). Data of IL-4 image analysis was also showing the same result (*p* < 0.01). Ctrl: normal feeding, HF: high-fat diet, HDE: high-fat diet and untreated AD-induced, HTT: high fat diet and Hataedock treated AD-induced, EP: epidermis, and DE: dermis. ^*∗∗*^ *p* < 0.01, compared with the Ctrl group; ^##^ *p* < 0.01, compared with the HF group; ^&&^ *p* < 0.01, compared with the HDE group.

The regulation of Th2 differentiation was estimated by measuring the IL-4-positive reaction. The IL-4-positive reaction was seen in the cytoplasm of dermal papilla cells. The levels of IL-4 in the HTT group were shown to be decreased by 54% (*p* < 0.01) as compared with the HDE group (Figure 3).

### 3.2. The Maintenance of Lipid Barrier in Epidermis

**Figure 4:**
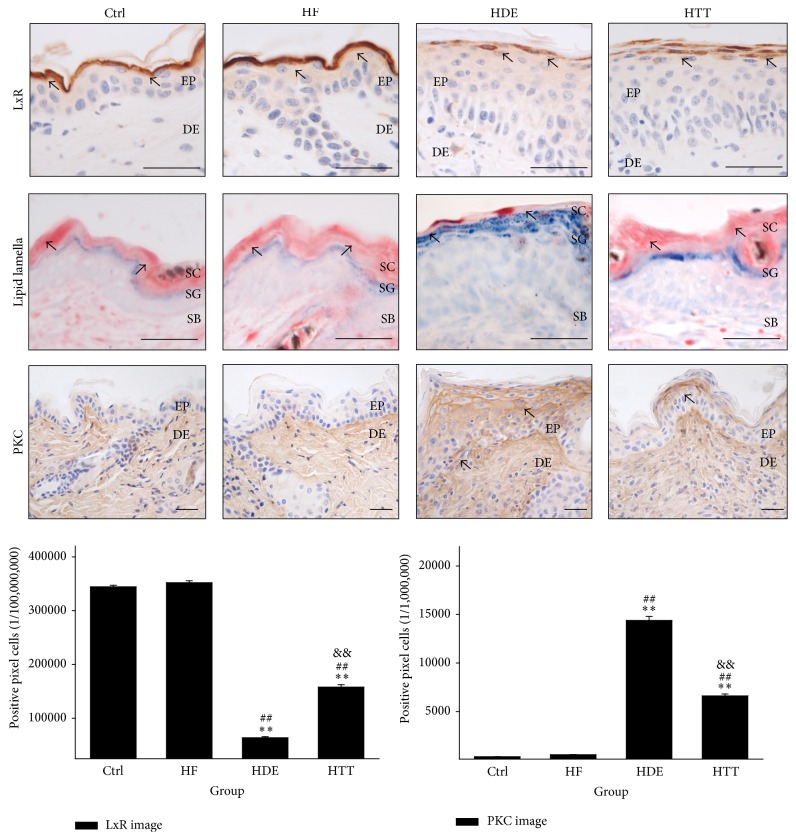
The maintenance of lipid barrier in epidermis. The LXR-positive reaction (arrow indicates dark brown) in HDE remarkably decreased but was maintained in HTT (LXR immunohistochemistry; bar size, 50 *μ*m). The intercellular lipid lamellae (arrow indicates reddish line) of the stratum corneum disappeared in HDE but appeared in HTT (oil red O; bar size, 50 *μ*m). The PKC-positive reaction (arrow indicates dark brown) in HTT remarkably decreased (PKC immunohistochemistry; bar size, 50 *μ*m). Data of LXR and PKC image analysis was also showing the same result (*p* < 0.01). Ctrl: normal feeding, HF: high-fat diet, HDE: high-fat diet and untreated AD-induced, HTT: high fat diet and Hataedock treated AD-induced, EP: epidermis, DE: dermis, SC: stratum corneum, SG: stratum granulosum, SB: stratum basale, LXR: liver X receptor, and PKC: protein kinase C. ^*∗∗*^ *p* < 0.01, compared with the Ctrl group; ^##^ *p* < 0.01, compared with the HF group; ^&&^ *p* < 0.01, compared with the HDE group.

The protective effects of the lipid barrier were estimated by measuring the liver X receptor- (LXR-) and PKC-positive reactions. The levels of LXR-positive reaction that were seen diffusely in the cytoplasm of cells in the stratum corneum and the stratum granulosum were remarkably decreased in the HDE group, but the levels of the HTT group were increased by 148% (*p* < 0.01) as compared with the HDE group (Figure 4).

An increase in the levels of PKC-positive reaction appearing in damaged keratinocytes and in the intercellular space was observed in the HDE group as compared with the Ctrl and HF group. This elevation was significantly decreased by HTD treatment. The levels of PKC in the HTT group were shown to be decreased by 54% (*p* < 0.01) as compared with the HDE group (Figure 4).

Moreover, we observed that skin damage, such as the elimination of the intercellular lipid lamellae in the stratum corneum, was remarkably reduced in the HTT group as compared with HDE group (Figure 4).

### 3.3. The Regulation of Mast Cells Activation

**Figure 5:**
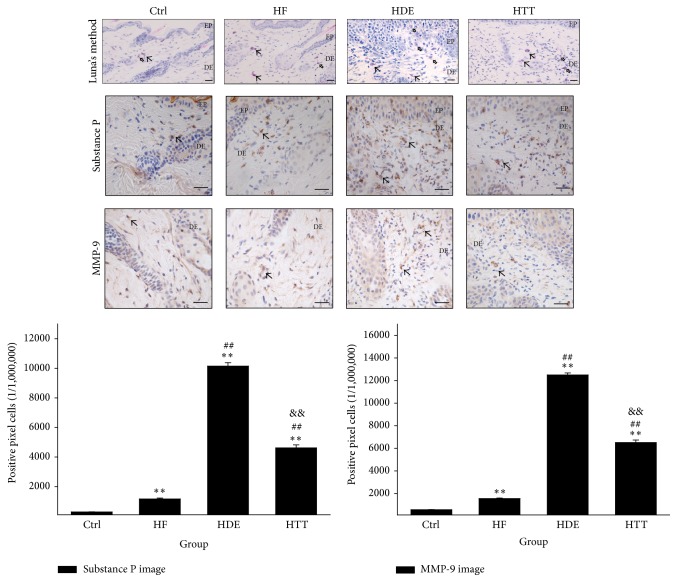
The regulation of mast cells activation. The distribution of degranulated mast cell (vacant arrow) in dermal papillae was increased in the HDE group but decreased in the HTT group (Luna's method; bar size, 50 *μ*m). The Substance P positive reaction (arrow indicates dark brown) in HTT significantly decreased (Substance P immunohistochemistry; bar size, 50 *μ*m). The MMP-9 positive reaction (arrow indicates dark brown) in HTT remarkably decreased (MMP-9 immunohistochemistry; bar size, 50 *μ*m). Data of Substance P and MMP-9 image analysis was also showing the same result (*p* < 0.01). Ctrl: normal feeding, HF: high-fat diet, HDE: high-fat diet and untreated AD-induced, HTT: high fat diet and Hataedock treated AD-induced, EP: epidermis, DE: dermis, and MMP-9: matrix metalloproteinases-9. ^*∗∗*^ *p* < 0.01, compared with the Ctrl group; ^##^ *p* < 0.01, compared with the HF group; ^&&^ *p* < 0.01, compared with the HDE group.

The regulation of mast cells activation was estimated by measuring the Substance P and matrix metallopeptidase 9- (MMP-9-) positive reaction in dermal papilla. Marked increases of Substance P and MMP-9-positive reactions, which were seen in the cytoplasm, were observed in the HDE group. Treatment with Hataedock suppressed the production of Substance P and MMP-9 significantly. The levels of Substance P in the HTT group were shown to be decreased by 54% (*p* < 0.01) as compared with the HDE group. The levels of MMP-9 in the HTT group were also shown to be decreased by 48% (*p* < 0.01) as compared with the HDE group (Figure 5).

Furthermore, the results of Luna's staining showed that many degranulated mast cells from the dermal papilla to the area around the subcutaneous layer were observed in the HDE group. On the other hand, decreases in degranulated mast cells were observed in the HTT group as compared with the HDE group (Figure 5).

### 3.4. Downregulation of Inflammation

**Figure 6:**
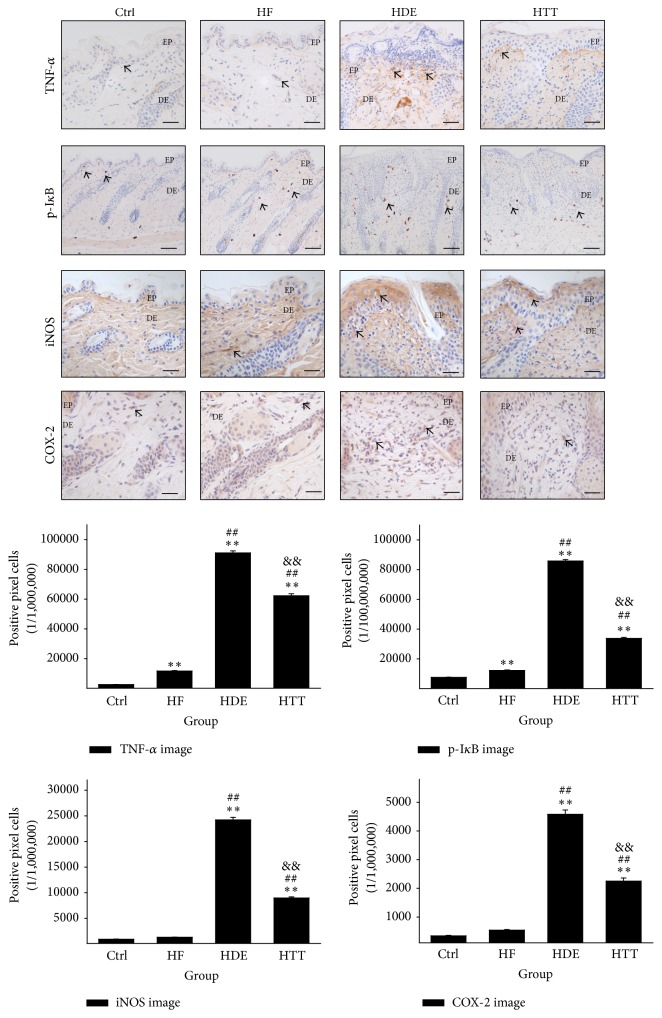
Downregulation of inflammation. In HTT group, the excessive inflammation condition such as increase of TNF-*α*, p-I*κ*B, iNOS, and COX-2 was ameliorated by HTD treatment. These positive reactions (arrow indicates dark brown) were remarkably decreased compared with those of the HDE group (immunohistochemistry; bar size, 50 *μ*m, only p-I*κ*B bar size 100 *μ*m). Data of TNF-*α*, p-I*κ*B, iNOS, and COX-2 image analysis was also showing the same result (*p* < 0.01). Ctrl: normal feeding, HF: high-fat diet, HDE: high-fat diet and untreated AD-induced, HTT: high fat diet and Hataedock treated AD-induced, EP: epidermis, DE: dermis. ^*∗∗*^ *p* < 0.01, compared with the Ctrl group; ^##^ *p* < 0.01, compared with the HF group; ^&&^ *p* < 0.01, compared with the HDE group.

To estimate the anti-inflammatory effects of HTD, we measured the levels of TNF-*α*-, p-I*κ*B-, iNOS-, and COX-2-positive reactions in stratum basale and dermal papilla. The results of the immunohistochemical staining showed the appearance of TNF-*α*-, p-I*κ*B-, iNOS-, and COX-2-positive reactions in the cytoplasm. Compared with the HDE group, HTD treatment significantly decreased the levels of TNF-*α*-, p-I*κ*B-, iNOS-, and COX-2-positive reactions. The HTT group showed a 32% (*p* < 0.01) decrease in TNF-*α* as compared with the HDE group. The HTT group showed a 61% (*p* < 0.01) decrease in p-I*κ*B as compared with the HDE group. The levels of iNOS in the HTT group were also shown to be decreased by 63% (*p* < 0.01) as compared with the HDE group. In addition, COX-2-positive reaction levels were decreased by 51% (*p* < 0.01) in the HTT group (Figure 6).

### 3.5. Upregulation of Apoptosis

**Figure 7:**
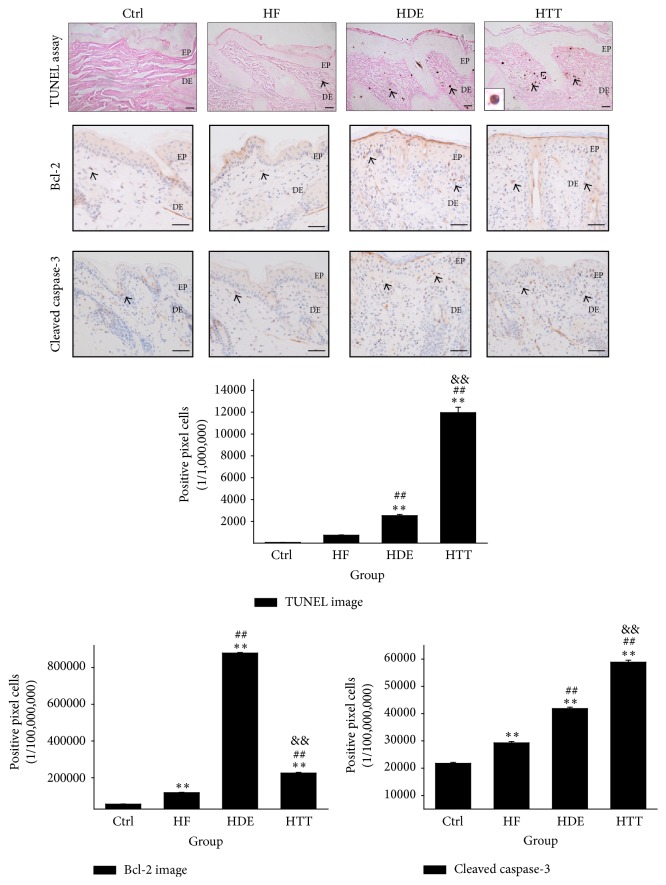
Upregulation of apoptosis. Upregulation of apoptosis in dermatitis induced by HTD treatment. The apoptotic body (arrow indicates dark brown) in the HTT group was remarkably increased compared to the HDE group (TUNEL assay; square box, enlarged DNA fragmentation of nucleus with TUNEL positive reaction; bar size, 50 *μ*m). The Bcl-2 positive reaction (arrow indicates dark brown) in HTT group was remarkably decreased compared with those of the HDE group (immunohistochemistry; bar size, 50 *μ*m). The cleaved caspase-3 positive reaction (arrow indicates dark brown) in HTT group was increased compared with those of the HDE group (immunohistochemistry; bar size, 50 *μ*m). Data of TUNEL, Bcl-2, and cleaved caspase-3 image analysis was also showing the same result (*p* < 0.01). Ctrl: normal feeding, HF: high-fat diet, HDE: high-fat diet and untreated AD-induced, HTT: high fat diet and Hataedock treated AD-induced, EP: epidermis, and DE: dermis. ^*∗∗*^ *p* < 0.01, compared with the Ctrl group; ^##^ *p* < 0.01, compared with the HF group; ^&&^ *p* < 0.01, compared with the HDE group.

The results of the TUNEL assay indicated the upregulation of apoptosis in the dermal papilla cells via the HTD treatment. In the HTT group, the apoptotic body as synonymous with gathering of DNA fragmentation on nucleus was remarkably increased by 373% (*p* < 0.01) as compared to the HDE group (Figure 7).

Compared with the HDE group, HTD treatment significantly changes the apoptosis signal. The HTT group showed a 74% (*p* < 0.01) decrease in Bcl-2, an antiapoptotic protein, as compared with the HDE group. The HTT group showed a 41% (*p* < 0.01) increase in cleaved caspase-3, proapoptotic protein, as compared with the HDE group (Figure 7).

### 3.6. The Mitigative Effect of HTD Treatment on Dermatosis

**Figure 8:**
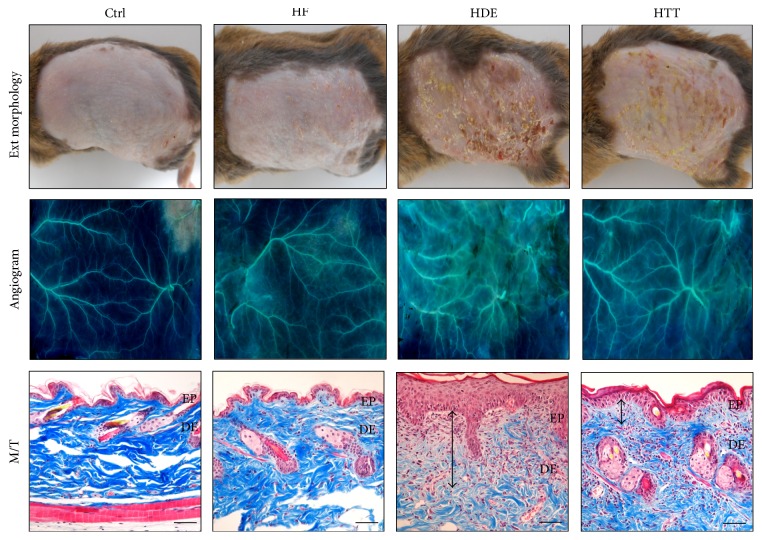
The mitigative effect of HTD treatment for dermatitis. The skin damage as eczema was mitigated in HTT group. The angiogenesis was increased in HDE group but decreased in HTT group (×4). The histological features of AD such as vacuolation of keratinocytes, hyperplasia, edema (up-down arrow), infiltration of inflammatory cells, and increase of capillary were increased in HDE group but decreased in HTT group (bar size, 100 *μ*m). Ctrl: normal feeding, HF: high-fat diet, HDE: high-fat diet and untreated AD-induced, HTT: high fat diet and Hataedock treated AD-induced, EP: epidermis, DE: dermis, and M/T: Masson trichrome method.

The therapeutic effectiveness of HTD (administering the extract of* Coptidis Rhizoma* and* Glycyrrhiza uralensis*) was explored by examining its effect on dermatosis severity. AD-induced mice that were fed a high-fat diet and did not receive any treatment (the HDE group) showed the highest level of dermatosis severity. The obtained image of the HDE group showed various pathological features, such as severe erythema, blood clot, edema, superficial erosion, deep excoriation, and dry skin. In contrast, the high-fat-diet-fed, AD-induced mice treated with HTD (the HTT group) exhibited better control of AD symptoms (Figure 8).

A comparison of angiogenesis was conducted between the HDE and HTT groups. Our angiogram reveals that the HTT group experienced much better angiogenesis reduction than the HDE group (Figure 8).

For the results obtained from Masson's trichrome stains, the atopic mice of the HDE group also exhibited significant damage such as vacuolation of keratinocytes, hyperplasia in epidermis, disappearance of collagen fibers, the infiltration of inflammatory cells, and an increase in dermal capillary density.

On the other hand, when mice were treated with HTD, the amelioration of epidermal hyperplasia, the improvement of collagen fiber density, and fewer inflammatory cell infiltrates in the dermis were remarkably observed (Figure 8).

## 4. Discussion

The results of the studies suggest that HTD treatment may be an effective preventive treatment for AD. HTD treatment was effective in attenuating inflammation and maintaining the skin barrier in AD-induced NC/Nga mice under high-fat-diet conditions. Much progress has been made in understanding the genetic background and pathophysiology of AD, thus allowing more specific therapeutic interventions to be introduced [23]. Among these interventions, we paid attention to those that intervene very early in the lives of infants and young children by controlling skin inflammation at the earliest time point [24]. Moreover, there is now plenty of evidence indicating close ties between the metabolic and immune systems. It is now clear that obesity is associated with a state of chronic low-level inflammation [25]. In this respect, an unbalanced maternal diet during breastfeeding may be a risk factor underlying the later development of atopic sensitization in the infant, regardless of maternal atopic disease [26]. It was also reported that neonatal adiposity is a predictor of AD [12].

We hypothesized that both immune system dysregulation and a high-fat diet may affect inflammatory responses in the infant. Further, we hypothesized that these responses may amplify the development of AD. Based on these hypotheses, we investigated the preventive therapeutic effects of HTD treatment on inflammatory regulation and skin protection in AD-induced NC/Nga mice under high-fat diet conditions. This study differs from previous studies with regard to the timing of treatment administration. Previous studies administered the herbal extracts after AD symptom onset [27–30]. On the other hand, this study administered the herbal extracts before clinical symptom onset. We also established a high-fat-diet-induced model and so attempted to identify relevant neonatal adiposity and the development of AD.

### 4.1. The Regulation of Th2 Differentiation

It was known that the Th2-biased immune responses that characterize neonates may influence the later onset of allergic disease [31]. Th2 cells mediate these functions by producing various cytokines. Particularly, IL-4 is a key Th2 cytokine that is critical for Th2 cell differentiation, IgE production, and eosinophil recruitment, among other functions [32]. It was reported that high levels of IL-4-producing T cells and low levels of IFN-*γ* at birth may enhance the risk of the subsequent development of AD [33].

To estimate the regulation of Th2 differentiation, we measured the level of IL-4-positive reaction in the dermal papilla cells. We show that the levels of IL-4 positive reaction were remarkably increased in the HDE group but that the levels of the HTT group were decreased (Figure 3). This finding suggests that HTD treatment reduced the level of IL-4. This decrease of IL-4 may contribute to the regulation of Th2 differentiation and these results may also contribute to the improvement of AD.

### 4.2. The Maintenance of Lipid Barrier in Epidermis

We show that the levels of LXR-positive reaction were remarkably decreased in the HDE group but that the level of the HTT group was maintained. Moreover, the lipid barrier in the intercellular space was also maintained in the HTT group. On the other hand, an increase in the levels of PKC was observed in the HDE group as compared with the Ctrl group. This elevation was significantly decreased in the HTT group (Figure 4).

It is known that dysfunctional ceramides in the SC barrier may contribute to the disruption of the epidermal barrier, resulting in mechanisms that operate in the pathophysiology of AD [34]. This condition is induced by PKC activation, which plays a role in the initiation of epidermal barrier dysfunction [35]. The PKC signaling pathway is activated by a broad spectrum of extracellular stimuli that promote lipid hydrolysis and play a fundamental role in numerous biological facets, such as differentiation, proliferation, apoptosis, and neuronal transmitter release [36]. It is known that transactivation by LXR was decreased by the activation of the PKC signaling pathway [37].

LXRs play a critical role in the control of lipid metabolism, acting as regulators of cholesterol and fatty acid metabolism [38]. Previous studies have demonstrated that LXR activators stimulate epidermal differentiation, improve permeability barrier homeostasis, and inhibit epidermal proliferation [39, 40]. Additionally, it is known that LXR activators exhibit potent antihyperplastic and anti-inflammatory activity in irritant-contact dermatitis and acute allergic-contact dermatitis [41]. LXR activation also accelerates permeability barrier recovery following acute barrier disruption [42].

Therefore, the protective effects for the lipid barrier may be caused by the activation of LXRs and the inhibition of PKC. Such effects may influence the maintenance of the lipid barrier in the HTT group.

### 4.3. The Regulation of Mast Cells Activation

Mast cells play a central role in both acute and chronic allergic reactions through the release of a number of mediators and cytokines [43]. Because most studies have shown increased numbers of mast cells in skin lesions in AD models, it is generally assumed that mast cells contribute to skin inflammation [2]. Upon activation, mast cells release their membrane-bound cytosolic granules, leading to the release of several molecules that are important in the pathogenesis of AD and host defense [44].

Mast cell associated nerves in the skin are predominantly Substance P-positive [45]. Substance P, an established neurotransmitter, evokes an immune inflammatory response involving the degranulation of mast cells [46]. Interestingly, it has also been pointed out that stress and anxiety worsen dermatitis via Substance P-dependent neurogenic inflammation in mice [47]. Thus, Substance P is currently considered to be one of the key pruritogenic factors [48].

Matrix metalloproteinase- (MMP-) 9 has been recognized in the process of inflammation and tissue remodeling and repair. Also, mast cells can produce MMP-9, which may contribute to extracellular matrix degradation and absorption in the process of allergic and nonallergic responses [49]. Thus, it is assumed that MMP-9 could be important in the pathogenesis of AD [50].

In the present study, many degranulated mast cells were observed in the HDE group. On the other hand, primarily granular mast cells appeared in HTT group. Moreover, the levels of Substance P and MMP-9 were significantly decreased in the HTT group (Figure 5). Therefore, these results imply that HTD reduces the infiltration of degranulated mast cells and can prevent the release of Substance P and MMP-9.

### 4.4. Downregulation of Inflammation

TNF-*α* is one inflammatory mediator that has been implicated in AD, due to its participation in lipid and protein synthesis in the epidermis and as a consequence of effect of skin barrier lipid composition and organization [51]. It is known that facilitated translocation of NF-*κ*B may worsen the allergic inflammation including AD by enhancing the production of inflammatory cytokines and chemokines [52]. And the phosphorylated I*κ*B (p-I*κ*B) allow the NF-*κ*B subunit to translocate to the nucleus [53]. For this reason, deregulation of NF-*κ*B and p-I*κ*B is a hallmark of chronic inflammatory diseases [54]. iNOS and COX-2 are also known to play important roles in the regulation of inflammatory reactions [55, 56]. iNOS produces high amounts of NO induced by cytokines such as interferon gamma and TNF-*α* [57]. NO derived from iNOS also plays important roles in the modulation of symptoms in patients with inflammatory diseases, including AD [58]. It is also known that COX products are increased in the skin of patients with AD [59].

To elucidate the role of the anti-inflammatory effects of HTD, we analyzed the levels of TNF-*α*-, p-I*κ*B-, iNOS-, and COX-2-positive reactions. Marked increases in TNF-*α*, p-I*κ*B, iNOS, and COX-2 production were observed in the HDE group. These increases were effectively lowered by HTD treatment in the HTT group (Figure 6). While the inflammatory response plays important roles in protecting the host and repairing tissues, it can also damage normal skin tissues. Therefore, these results of the decrease in inflammatory cytokines imply that HTD may contribute to the reduction of inflammation and improvement of AD.

### 4.5. Upregulation of Apoptosis

Apoptosis is highly important in the renewal of cells and formation of epidermal structure [60]. Apoptotic cells may not stimulate inflammation if they are ingested by phagocytes before they release their intracellular substances. Moreover, during this process apoptotic cells can stimulate phagocytes to induce anti-inflammatory cytokines [61]. It has also been reported that dysregulated apoptosis may contribute to the development and persistence of AD [62].

Overexpression of the Bcl-2 has been demonstrated to prevent apoptosis either by sequestering proforms of death-driving caspases or by preventing the release of cytochrome c into the cytoplasm [63, 64]. On the other hand, sequential activation of caspases plays an important role in the execution-phase of cell apoptosis. Among them, caspase-3 is a commonly activated death protease, catalyzing the specific cleavage of many key cellular proteins [65]. The caspase-3 is known to act downstream on bax/bcl-2 control [66]. The cleavage products of bcl-2 are located to the mitochondria resulting in the release of cytochrome c and leading to more caspase-3 activation as a positive feedback effect, strengthening the apoptotic effect [67].

In the present study, apoptotic cells were determined via TUNEL assay. The apoptotic bodies in the HTT group were remarkably increased in number as compared to the HDE group. The cleaved caspase-3 positive reaction was also increased in the HTT group. In contrast, the Bcl-2 positive reaction in HTT group was remarkably decreased (Figure 7). This finding suggests that HTD treatment may contribute to stimulate the apoptotic cascade activation that ultimately leads to improvement of AD.

### 4.6. The Mitigative Effect of HTD Treatment on Dermatosis

In this study, we demonstrated the skin barrier maintenance and anti-inflammatory effects of HTD treatment under high-fat-diet conditions in AD. In our animal model, the repeated application of DNFB caused skin damage and increased angiogenesis and spongiosis in the HDE group. However, in the HTT group, damage to the intercellular space, hyperplasia, edema, the infiltration of inflammatory cell, and increased capillaries were decreased (Figure 8).

Therefore, these findings suggest that HTD was more effective in maintaining skin integrity during the course of dermatosis and treatment under high-fat-diet conditions. These results indicate that HTD may alleviate the underlying inflammatory reactions and effectively attenuate these skin damages.

## 5. Conclusions

In summary, we demonstrated that HTD treatment was effective in attenuating inflammation and maintaining the skin barrier in AD-induced NC/Nga mice under high-fat-diet conditions. These results imply that HTD may alleviate the symptom of AD through the regulation of Th2 differentiation, the maintenance of lipid barrier in epidermis, the regulation of mast cells activation, downregulation of inflammation, and upregulation of apoptosis. In conclusion, HTD could be used as an alternative and preventive therapeutic approach in the management of AD. Further studies about detailed mechanism of these protective immune responses are needed.
